# Telemedicine impact on patient disparities and physician practice patterns in cancer rehabilitation: A multicenter retrospective study

**DOI:** 10.1002/pmrj.13199

**Published:** 2024-06-12

**Authors:** David Leong, Amy Ng, Philip Chang, Jasmine Zheng, Richard Wilson, Matthew Edwin Chen, Mary Vargo

**Affiliations:** ^1^ Department of PM&R Case Western Reserve University/MetroHealth Rehabilitation Institute Cleveland Ohio USA; ^2^ Department of Palliative Rehabilitation and Integrative Medicine MD Anderson Cancer Center Houston Texas USA; ^3^ Department of PM&R Cedars‐Sinai Los Angeles California USA; ^4^ Department of PM&R University of Pennsylvania Philadelphia Pennsylvania USA

## Abstract

**Objective:**

To examine the impact of telemedicine on demographic and practice patterns between outpatients receiving virtual versus in‐person cancer rehabilitation physiatry care.

**Design:**

Multicenter retrospective study.

**Setting:**

Outpatient cancer rehabilitation physiatry clinics at four academic medical centers in the United States.

**Patients:**

Patients with cancer diagnoses or history of cancer diagnosis.

**Interventions:**

Cancer rehabilitation physiatry encounters.

**Main Outcome Measures:**

Visit mode (in‐person, telemedicine); disparities variables (age, race, and gender) by visit mode, and practice interventions (imaging, medications, procedures, other orders, and orders of any type) by visit mode.

**Results:**

Among a total of 7004 encounters, 2687 unique patients were found. In‐person participants were significantly older than the average telemedicine participant (mean 62.9 vs. 60.7 years; *p* < .001). A race effect was seen (*p* = .037) with individuals reporting as Asian or other being more likely to have telemedicine encounters. No gender disparities were seen. Using a random visit analysis model to compare populations receiving in‐person versus telemedicine care, a slight majority (53%) of follow‐up visits were via telemedicine, versus 40% of new patient visits (*p* < .001). No significant differences were seen in medication prescribing frequency (38.9% telemedicine vs. 36.7% in‐person, adjusted relative risk [RR]: 0.988, confidence interval [CI]: 0.73–1.34; *p* = .988) or imaging frequency (2.4% telemedicine vs. 7.6%; adjusted RR: 0.784, CI: 0.44–1.39; *p* = .408) between telemedicine versus in‐person visit types. Other orders were significantly less likely to be placed during telemedicine than in‐person visits (19.9% telemedicine vs. 28.6% in‐person; adjusted RR: 0.623, CI: 0.45–0.86, *p* = .004). Order(s) of any type were placed in 54% of visits (52% telemedicine vs. 56% in‐person; adjusted RR: 0.92 for telemedicine, CI: 0.83–1.01, *p* = .082).

**Conclusions:**

Telemedicine has been integrated into cancer rehabilitation physiatry practices and appears to be conducive for placing many types of orders, especially medications. Age was found to be the only major demographic difference between in‐person and telehealth patients.

## INTRODUCTION

In response to the COVID‐19 pandemic, health care made a sudden shift to telemedicine visits to provide continued care in a safer environment for both patients and practitioners. Within physiatry, telemedicine has been used for supervising therapies,^1^, ^2^, ^3^ addressing common impairments seen in conditions including stroke,^2^ spinal cord injury, and multiple sclerosis,^4^, ^5^ as well as for reducing barriers to health care access.^6^ Even before the pandemic, cancer rehabilitation telehealth had begun to espouse a wide variety of approaches including telephone, video, and asynchronous interventions such as activity tracking, internet portal availability, mobile health apps, and instant messaging, as well as various models including hybrid (combined in‐person and telemedicine), interdisciplinary collaborative, and virtual reality designs.^7^, ^8^, ^9^, ^10^ Specific areas of clinical focus have included psychotherapy; aspects of skilled physical, occupational, and speech therapies; exercise interventions; symptom management; and education and support groups.^7^, ^11^

Within cancer rehabilitation physiatry specifically, during‐pandemic survey data have shown high levels of satisfaction among both patients and providers.^12^ Of note, patient satisfaction has also been demonstrated with telemedicine for general outpatient cancer care.^13^ Areas empirically proposed as most conducive for telemedicine intervention in cancer rehabilitation physiatry practices are those typically less heavily dependent on physical examination and include cancer‐related fatigue, follow‐up for stable problems, education and counseling, medication rotation/titration, preoperative counseling, and cancer‐related cognitive impairment.^14^

Speculative risks have been expressed of telemedicine in general, including potential for overutilization of testing in place of physical examination, and interference with evidence‐based care.^15^ Although widespread cost effectiveness data are still lacking, the Collaborative Care to Preserve Performance in Cancer (COPE) trial demonstrated favorable cost effectiveness of a telerehabilitation model compared with usual care in an advanced cancer population.^16^ However, real‐world clinical practice patterns with regard to cancer rehabilitation physiatry telemedicine versus in‐person visit contexts have not yet been described.

Access to health care is a social determinant of health of particular concern for cancer rehabilitation patients, given that most cancer rehabilitation providers practice at tertiary centers, where only a small minority of patients receive their cancer care.^17^ Although an advantage of telemedicine includes potential improved access with regard to geographic or transportation barriers, concern also exists for widening disparities based on gaps in access to the necessary communication technologies.^11^ Specific concerns include the impact of disability or language barrier in accessing telemedicine platforms and ramifications of any barriers for high‐risk populations, including minority groups.^18^ Patients with cancer may have clinical vulnerabilities different from other rehabilitation cohorts, especially during the active treatment phase. Additionally, although beyond the scope of this article to summarize, cancer epidemiology studies continue to report underlying racial and socioeconomic disparities within the cancer population, including cancer treatment outcomes.^19^ As with clinical practice patterns, the actual impact of telemedicine versus in‐person cancer rehabilitation physiatry practice regarding disparities issues has received limited attention.

This retrospective multicenter study aims to identify if there are differences in physician clinical practice patterns between in‐person and telemedicine outpatient cancer rehabilitation physiatry visits. Additionally, evaluation for potential disparities is conducted between the populations who receive telemedicine and in‐person care.

## METHODS

Retrospective data were gathered from four academic outpatient cancer rehabilitation physiatry clinics. A data use agreement between all four institutions was acquired before sharing information, with each institution pseudonymizing participant identifiers. Patients 18 years or older were included, with outpatient visit dates between March 23, 2020 and August 31, 2021, and with a cancer diagnosis coded in the encounter. All non‐cancer‐related and procedure‐only encounters were excluded. Participant demographic data (age, gender, race), encounter type (new versus follow‐up, in‐person versus telemedicine, phone vs. video), and physician orders (medication, imaging, procedures, other orders, and whether any order was placed) were collected. Physician order data were assessed in a binary fashion per encounter, rather than total quantities. For example, an encounter where two medications and three x‐rays were ordered would count as one for each category: medications and imaging. Imaging orders included plain x‐rays, ultrasound studies, computed tomography, magnetic resonance imaging, nuclear medicine, or other imaging studies. Procedure orders included referrals for procedures; procedures done in office were not included as there was no telemedicine equivalent. The “other” category included a heterogeneous mix of orders including rehabilitation therapy referrals, subspecialty consultations, home care, durable medical equipment and supplies, and laboratory data.

A random encounter was extracted from each unique participant to create two independent groups for the comparison of the effect of telemedicine compared with in‐person visits. By assigning only one encounter per unique participant, this random visit analysis avoids complications of longitudinal data such as complex correlation structures, irregularly spaced visits, missing data, and mixtures of time‐varying and static covariate effects.^20^ In real practice, after a single new encounter, a patient may have many follow‐up visits. Within the cancer population, the most complex visit may not always occur at the first encounter. Additionally, with the time frame of the study, data may have been collected in the middle of care, so not all patients may have had a new visit. These issues informed the rationale for selecting a random rather than new or “earliest” visit for any particular patient. Univariate and adjusted analyses are presented. Longitudinal (total encounter) data are presented descriptively. Other order data are also compiled descriptively, from the total encounter data set, as well as chi‐square analyses performed for in‐person versus telemedicine order frequency for the most common “other” order types in the longitudinal data set.

The random visit analysis was performed with a generalized linear mixed model that estimated the effect of a telehealth visit versus in‐person visit on physician orders with covariates for age, race (White, Black, Asian, other), visit type (new vs. follow‐up), and gender (female vs. male). A Poisson probability model was chosen given that the outcome data are binary and a log‐link function was applied. To adjust for differences between institutions, the models included hierarchical random effects of participants nested within institutions. The adjusted relative risks (RRs) of physician orders during telehealth visits are presented. The model for physician orders of Procedures was not able to be estimated due to the infrequent occurrence in telehealth and in‐person visits when considering a single visit per person. In this case, the unadjusted relative risk‐based and chi‐square hypothesis tests are presented. Unadjusted comparisons included *t*‐tests for continuous data and chi‐square tests for categorical data. All statistical analyses were performed with SAS/STAT software, Version 9.4 (SAS Institute Inc., Cary, NC, USA).

## RESULTS

The total sample contained 7004 encounters with 2687 unique patients (Table 1). A total of 3746 (53.5%) encounters were in person and 3258 (46.5%) were via telemedicine. Video visits were the predominating telemedicine format in three of the four institutions and occurred in 2523 visits overall (77% of all telemedicine encounters). Orders of any type were placed in more than half of all encounters (55.7%) with the most common type of order being for medication, occurring in 43% of encounters, followed by other orders (20.5%). Imaging (4.2%) and procedure (3.5%) orders were much less common (Table 2). Of the other orders (Table 3), most frequent were physical therapy (10.4%), case management or social work (5.5%), laboratories (5.1%), and occupational therapy (4.1%). Physical therapy, occupational therapy, lab, and durable medical equipment orders were more frequent with in‐person than telemedicine visits, and social work or case management referrals were more frequent with telemedicine (Table 4, data available for institutions 1, 2, and 4).

**TABLE 1:** Individual institution visit type data (total encounters).

Institution 1 *n* = 627				Institution 2 *n* = 3985				Institution 3 *n* = 1638				Institution 4 *n* = 754			
IP	TM	Phone	Video	IP	TM	Phone	Video	IP	TM	Phone	Video	IP	TM	Phone	Video
540 (86.1)	87 (13.9)	78 (12.4)	9 (1.4)	1957 (49.1)	2028 (50.9)	503 (12.6)	1525 (38.3)	750 (45.8)	888 (54.2)	135 (8.2)	753 (46.0)	499 (66.2)	255 (33.8)	19 (2.5)	236 (31.3)
		90%	10%			25.0%	75.0%			15.2%	84.8%			7.5%	92.5%

Abbreviations: IP, in person; TM, telemedicine.

**TABLE 2:** Longitudinal (total encounter) sample demographics and primary outcomes.

Parameter	Total sample *N* = 7004 (%)	Institution 1 *N* = 627 (9.0%)	Institution 2 *N* = 3985 (56.9%)	Institution 3 *N* = 1638 (23.4%)	Institution 4 *N* = 754 (10.8%)
Age (SD)	63.1 (12.4)	62.9 (11.0)	64.2 (12.6)	59.5 (13.7)	57.7 (13.0)
Male (%)	2847 (41)	216 (34.4)	1949 (48.9)	436 (26.6)	246 (32.6)
Female (%)	4157 (59)	411 (65.6)	2036 (51.1)	1202 (73.4)	508 (67.4)
Asian (%)	334 (4.8)	17 (2.7)	177 (4.4)	116 (7.1)	24 (3.2)
Black (%)	1179 (16.8)	249 (39.7)	515 (12.9)	134 (8.2)	281 (37.3)
White (%)	4926 (70.3)	328 (52.3)	2930 (73.5)	1238 (75.6)	430 (57.0)
Other (%)	565 (8.1)	33 (5.3)	363 (9.1)	150 (9.2)	19 (2.5)
New patient visits	2201 (31.4)	201 (32.1)	1391 (34.9)	411 (25.1)	198 (26.3)
Follow‐up patient visits	4803 (68.6)	426 (67.9)	2594 (65.1)	1227 (74.9)	556 (73.7)
Medication	3050 (43.5)	220 (35.1)	1690 (42.4)	712 (43.5)	428 (56.8)
Imaging	296 (4.2)	89 (14.2)	56 (1.4)	34 (2.1)	117 (15.5)
Procedure	296 (3.5)	18 (2.9)	139 (3.5)	73 (4.5)	16 (2.1)
Other	1434 (20.5)	319 (50.9)	598 (15.0)	182 (11.1)	335 (44.4)
Any order	3903 (55.7)	439 (70.0)	2016 (50.6)	852 (52.0)	596 (79.1)

**TABLE 3:** Other orders by institution (all encounters).

Other orders for all visit types (IP + TM)	Total *N* = 7004 (%)	Institution 1 *N* = 627	Institution 2 *N* = 3985	Institution 3 *N* = 1638	Institution 4 *N* = 754
Physical therapy	732 (10.4)	184 (29.3)	170 (4.3)	96 (5.9)	282 (37.4)
Occupational therapy	285 (4.1)	16 (2.6)	84 (2.1)	38 (2.3)	147 (19.5)
Speech therapy	31 (0.44)	8 (1.3)	11 (0.27)	8 (0.49)	4 (0.53)
Home care	60 (0.86)	24 (3.8)	19 (0.48)	10 (0.61)	7 (0.93)
Case management, social work	383 (5.5)	25 (4.0)	300 (7.5)	17 (1.0)	41 (5.4)
Nutrition	42 (0.91)	12 (1.9)	30 (0.75)	–	–
Laboratories^a^	359 (5.1)	96 (15.3)	62 (1.6)	54 (3.3)	147 (19.5)
DME	189 (3.5)	157 (25)	5 (0.13)	–	27 (3.6)
Neuropsychology		–	–	17 (1.0)	–

Abbreviations: DME, durable medical equipment; IP, in person; TM, telemedicine.

^a^ All laboratories (may be more than one per encounter).

**TABLE 4:** Other orders by visit type (all encounters).

	Total *N* = 5366 (%)	In person *N* = 2995	Telemedicine *N* = 2368	*p* value (IP vs. TM)	*Video N* = 1769	Phone *N* = 599
Physical therapy	636 (11.8)	470 (16.0)	166 (7.0)	<.001	141 (7.9)	25 (4.2)
Occupational therapy	247 (4.6)	173 (5.8)	74 (3.1)	<.001	63 (3.6)	11 (1.8)
Speech therapy	23 (0.43)	16 (0.53)	7 (0.30)	.139	6 (0.34)	1 (0.16)
Home care	50 (0.93)	30 (1.0)	20 (0.84)	.353	14 (0.79)	6 (1.0)
Case management, social work	367 (6.8)	142 (4.7)	225 (9.5)	<.001	178 (10.0)	47 (7.8)
Nutrition	42 (0.78)	23 (0.76)	19 (0.80)	.887	13 (0.73)	6 (1.0)
Laboratories^a^	258 (4.8)	198 (6.6)	60 (2.5)	<.001	46 (2.6)	14 (2.3)
Durable medical equipment	189 (3.5)	167 (5.6)	22 (0.92)	<.001	9 (0.51)	13 (2.2)

*Note*: Data available for institutions 1, 2, and 4.

Abbreviations: IP, in person; TM, telemedicine.

^a^ All laboratories (may be more than one per encounter).

### Random Visit (Unique Patient) Visits: Univariate and Adjusted Analyses of Population Characteristics and Clinical Interventions

The population of unique patients was majority (61%) female, with average age 61.7 years, and race demographics including 70% White, 19% Black, 4% Asian, and 7% other (Table 5). Telemedicine encounters were significantly more likely to occur as a follow‐up compared to a new patient visit, with 62.4% of telemedicine visits being follow‐ups, whereas in‐person visits were evenly divided between new (50.4%) and follow‐up (49.6%) encounters (*p* < .001 for the difference between in‐person and telemedicine visits with regard to new versus follow‐up encounter status). (Table 5) No differences between in‐person and telemedicine visits were seen with regard to gender. In‐person participants were significantly older compared to the average telemedicine participant (62.9 vs. 60.7 years; *p* < .001). An effect of race was seen with patients identifying as Asian (56%) or other (52%) being more likely to have telemedicine visits, and those identifying as Black (47%) or White (47%) being less likely to have telemedicine visits (*p* = .037). In the unadjusted analysis, medications were the most frequent type of order for both in‐person (36.7%) and telemedicine (38.9%) encounters, not significantly different between the two visit types. The frequency of placing any order during an encounter was 56% for in person and 52% for telemedicine (*p* = .033). Both imaging (2.4% vs. 7.6%; *p* < .001) and other (19.9% vs. 28.6%; *p* < .001) orders were significantly less likely to occur during a telemedicine encounter compared to in‐person visit. Procedure orders showed no difference between in‐person (3.8%) and telemedicine (2.6%) settings (*p* = .085). In the adjusted analysis (Table 6), other orders (RR: 0.623; *p* = .004) remained less frequent with telemedicine, medication prescribing frequency continued to show no difference between telemedicine and in‐person visits (RR: 0.998; *p* = .937), and imaging (RR: 0.784; *p* = .408) and any order frequency (0.848; *p* = .175) became nonsignificant between telemedicine and in‐person settings. Due to insufficient numbers, a reliable adjusted analysis could not be obtained for procedure orders.

**TABLE 5:** Random visit (unique patient) unadjusted analysis demographics.

Parameter	In person *N* = 1424	Telemedicine *N* = 1263	*p* value
Age (SD)[M]	62.9 (13.0) [65]	60.7 (13.7) [63]	<.001^a^
Gender			.366^b^
Male (%) *N* = 1048 (39)	544 (38.2)	504 (39.9)	
Female (%) *N* = 1639 (61)	880 (61.8)	759 (60.1)	
Race			.037^b^
Asian (%) *N* = 136 (5)	60 (4.21)	76 (6.02)	
Black (%) *N* = 471 (18)	265 (18.6)	206 (16.3)	
White (%) *N* = 1862 (69)	994 (69.8)	868 (68.7)	
Other (%) *N* = (8)	105 (7.37)	113 (8.95)	
Visit type			<.001^b^
New (%) *N* = 1194 (44)	718 (50.4)	476 (37.7)	
Follow‐up (%) *N* = 1493 (56)	706 (49.6)	787 (62.4)	
Order type			
Imaging	109 (7.6)	30 (2.4)	<.001^b^
Medications	523 (36.7)	491 (38.9)	.252^b^
Procedures	54 (3.8)	33 (2.6)	.085^b^
Other	408 (28.6)	252 (19.9)	<.001^b^
Any order	798 (56.0)	656 (51.9)	.033^b^

^a^ Independent samples *t*‐test.

^b^ Chi‐square test.

**TABLE 6:** Random visit (unique patient) adjusted relative risks of primary outcomes.

Outcome	Adjusted^a^ relative risk (95% CI)	*p* value
Imaging	0.78 (0.44–1.40)	.409
Medications	0.98 (0.72–1.33)	.880
Procedures	0.68 (0.44–1.06)^b^	.085
Other	0.62 (0.45–0.86)	.004
Any order	0.84 (0.67–1.08)	.176

Abbreviation: CI, confidence interval.

^a^ Relative risk of physician orders in telemedicine encounters as compared with in‐person encounters, adjusted for age, gender, and race of the patient and whether the visit was new or follow‐up.

^b^ The unadjusted relative risk and hypothesis testing with a chi‐square test.

## DISCUSSION

The present findings demonstrate that telemedicine was integrated into cancer rehabilitation physiatry care during the COVID‐19 pandemic, with almost half the encounters in this sample being via telemedicine. Regarding disparities, an age effect was seen with the average age of patients receiving telemedicine visits being younger than those receiving in‐person visits (62.5 years for in person and 60.3 years for telemedicine). Other studies, across a wide range of patient populations, have noted age‐related differences in adoption of telemedicine^21^, ^22^, ^23^, ^24^, ^25^, ^26^ due to technological barriers in older individuals, patient preference,^27^ and confidentiality concerns.^28^ No gender‐related disparities were seen in our study. Race showed a significant effect in this study, with Asian and other groups showing greater likelihood of telemedicine visits than Blacks and Whites. Other studies have been mixed in whether race effects were seen, with some showing less telemedicine use in non‐White patients^21^ and others not.^25^ In a large sample of more than 2 million pandemic‐era encounters across multiple medical specialties, Hsiao et al.^29^ noted that within most specialties, male patients and patients of non‐White race were less likely to use telemedicine, but differences between specialties were noted. The Hsiao study also noted increased telemedicine use among rural patients. Although this was beyond the scope of what we were able to evaluate, the question does pertain to cancer rehabilitation medicine, which tends to be located in quaternary urban centers and with patients sometimes residing at considerable distances.

Regarding technological access in older individuals, Lu's study of 9185 Medicare beneficiaries found that nearly 85% of patients reported having internet access, with certain specific factors associated with lower rates of access, most strikingly that 50% of individuals with dual Medicare/Medicaid eligibility reported lack of internet availability, as well as significantly less internet availability seen in older age (74+) individuals, non‐Whites, non‐Metro geographic residents, and among those with certain health conditions.^30^ Notably though, in this Medicare study, cancer was not a health condition showing a difference in internet access compared with the average.

Regarding clinical practice patterns, new patient visits were more likely to occur with in‐person than telemedicine visits, and, although that is an expected finding, it is nonetheless noteworthy that many new patient visits (38% of all new patient visits per random visit analysis) were conducted by telemedicine. With respect to clinical interventions, per adjusted analysis, the following overall patterns emerged: (1) medication prescribing patterns showed no difference between in‐person and telemedicine visits; (2) imaging, procedure, and any ordering were also not significantly different between in‐person and telemedicine settings; and (3) other orders were less likely to be placed with telemedicine.

From an overall clinical practice standpoint, various observations can be made. Contrary to initial speculative concerns, imaging was not ordered more frequently during telemedicine encounters compared to in person. The lack of difference between in‐person and telemedicine encounters with regard to frequency of medications being prescribed was especially striking and supports the utility of telemedicine for this clinical context. Telemedicine visits are also likely a satisfactory format for many or most other types of orders. Although a formal cost analysis was beyond the scope of this article, there is no indication of higher costs being associated with telemedicine.

Also of note, although percentages varied, in general, the practice pattern trends were consistent between institutions, with medications being the most frequent individual order type across most institutions (exception of institution 1, where other orders were more frequent), and with imaging and procedure orders much less frequent than seen in the other categories. An order of any type was placed in at least 50% of visits across all institutions. Furthermore, the data reflect the heterogeneity of cancer rehabilitation medicine practice, with a variety of order types seen.

For the telemedicine encounters, factors driving telephone versus video formats were not assessed, though, empirically, the fact that current reimbursement structures favor video visits was likely influential, with telephone reimbursement, on a time basis, limited to 30 minutes.^31^ Survey data also support that most cancer rehabilitation physiatrists prefer video over telephone visits.^12^ Of note, however, institution 1 of our sample was a safety net hospital with many patients lacking reliable internet access, and that institution had the lowest percentage of telemedicine visits (14.1%), and of the telemedicine visits conducted there, most were telephone encounters (90%), suggesting a potentially important role for maintaining the option of telephone access, as a matter of policy, from a disparities standpoint. In our study, as noted, age was the major disparity seen; multiple studies have shown a predilection for telephone over video visits in older individuals,^23^, ^26^, ^30^ though this can be mitigated by other factors such as familiarity with technology.^23^

The strengths of this study lie in the multicenter design with a large sample size, geographic diversity, and overall racial demographics similar to population estimates from 2020 U.S. census data^32^ (White: 75.5%, Black: 13.6%, Asian: 6.3%), albeit with variations between institutions. Additionally, this novel data set presents information about some aspects of the clinical practice of physiatrists in cancer rehabilitation. For example, it was observed that typical care includes medications being ordered in 43% of visits and any order in 53% of visits.

### Limitations

This was a retrospective study. Both patient and physician bias influenced the decision to choose one encounter type over the other, as well as institutional policies. Patients may have limited telecommunication access or technologic literacy, or they may have a preference for in person. Physicians may want to perform specific physical examinations in person or anticipate a straightforward medication refill encounter that would be feasible via telemedicine. However, the extent to which physical examination was performed, or deemed necessary to perform, during telemedicine encounters (or even with in‐person encounters for that matter), was beyond the scope of this study. Cancer rehabilitation physiatry outpatient care can be highly varied and identification of the exact clinical reasons for the visits was also beyond the scope of the study.

Regarding the clinical practice data, there could be concern of skew in the findings, from such factors as the COVID‐19 pandemic, with potential bias against testing or imaging, especially in the early pandemic months, as well as from interinstitutional differences, with institution 2 comprising 57% of the total sample. Regarding interinstitutional observations, for example, imaging was ordered in 1.4% of visits at institution 2, compared to about 15% of the visits at institutions 1 and 4 (though institution 3, at 2.1%, was more similar to 2). The factors affecting interinstitutional differences are beyond the scope of being identifiable within these data but could relate to patient mix, institutional culture, and protocols, as well as individual physiatrist practice variation. Reassuringly, although our original methodology did not adjust for possible institutional confounders, when we reconfigured our random visit analysis to include adjustment for institution, with results as seen in Table 6, our findings were nearly identical to the original analysis, indicating negligible effect of institutional bias on the measured outcomes, not affecting interpretation.

Some socioeconomic factors, such as income disparities and more detailed information on ethnicity (for example, Hispanic/Latino individuals), were not able to be captured during this study and may contribute to disparity. From an economic standpoint, we considered including zip code as a proxy for income, but there were significant concerns with such an approach including that available zip code information at the time of the data analysis was not recent enough to be reliable and also that in at least one of our centers many patients travel significant distances for their care, with potential for confusion in the data between patients' home and hospital‐proximity zip codes. We also considered using health insurance type as a marker of economic status, but this was not feasible due to differences in data formatting between centers. Although geographic diversity is a strength, we have not assessed for more‐in‐depth factors as urban versus rural representation. Timeline data (such as “phase” of pandemic), which could have a significant impact on results, were not feasible to incorporate, nor were the current data yet able to be compared to practice patterns during a nonpandemic period. Clinical outcomes of telemedicine versus in‐person care were not assessed. Additionally, cost‐effectiveness data of telemedicine compared with in‐person care were not examined, nor were strategies to overcoming disparities and barriers to telemedicine.

Regarding the other order category, given the retrospective design, data were extracted from four slightly different EPIC^33^ versions, leading to possible inconsistencies. Over 40 variables were collected in the other category. The other category includes interventions of tremendous relevance in physiatry care, including rehabilitation therapy referrals, subspecialty physician consults, home health care, laboratory data, and orders for durable medical equipment and other supplies. The most common other referrals in this sample were physical therapy, laboratories, case management/social work, and occupational therapy. Qualitatively, substantial interinstitutional differences appear to be present, likely a combination of genuine differences and electronic data capture variances. Another substantial information gap with regard to clinical practice includes clinician efforts that are unlikely to be captured with an electronic order, such as education and counseling, interdisciplinary communication, form completion, and letters.

## CONCLUSIONS

Telemedicine has been integrated into cancer rehabilitation practice. Within this sample, almost half of all encounters were via telemedicine. Age was the major disparity found to be associated with telemedicine and in‐person encounter types. Medication management was the most frequent order placed by physicians during telemedicine encounters, similar in frequency to that occurring during in‐person visits. Areas of focus for future study can include optimizing integration of telemedicine into cancer rehabilitation care, such as promoting consistent accessibility, evaluating specific clinical scenarios and treatment models for best clinical outcomes and cost effectiveness, and exploring use of telemedicine to link with community centers where a majority of cancer care is provided.

## DISCLOSURES

The authors report no conflicts of interest.
